# Non-motor symptoms in amyotrophic lateral sclerosis

**DOI:** 10.1080/21678421.2023.2263868

**Published:** 2024-01-23

**Authors:** Ali Shojaie, Ahmad Al Khleifat, Sarah Opie-Martin, Payam Sarraf, Ammar Al-Chalabi

**Affiliations:** 1 Maurice Wohl Clinical Neuroscience Institute, Institute of Psychiatry, Psychology and Neuroscience, King’s College London, London, UK; 2 Department of Neuromuscular Diseases, Iranian Centre of Neurological Research, Neuroscience Institute, Tehran University of Medical Sciences, Tehran, Iran, and; 3 Department of Neurology, King’s College Hospital, London, UK

**Keywords:** Amyotrophic lateral sclerosis, non-motor symptoms, questionnaire, cold limbs, pain

## Abstract

**Objective:**

While motor symptoms are well-known in ALS, non-motor symptoms are often under-reported and may have a significant impact on quality of life. In this study, we aimed to examine the nature and extent of non-motor symptoms in ALS.

**Methods:**

A 20-item questionnaire was developed covering the domains of autonomic function, sleep, pain, gastrointestinal disturbance, and emotional lability, posted online and shared on social media platforms to target people with ALS and controls.

**Results:**

A total of 1018 responses were received, of which 927 were complete from 506 people with ALS and 421 unaffected individuals. Cold limbs (p 1.66 × 10^−36)^, painful limbs (p 6.14 × 10^−28^), and urinary urgency (p 4.70 × 10^−23^) were associated with ALS. People with ALS were more likely to report autonomic symptoms, pain, and psychiatric symptoms than controls (autonomic symptoms *B* = 0.043, p 6.10 × 10^−5^, pain domain B = 0.18, p 3.72 × 10^−11^ and psychiatric domain *B* = 0.173, p 1.32 × 10^−4^).

**Conclusions:**

Non-motor symptoms in ALS are common. The identification and management of non-motor symptoms should be integrated into routine clinical care for people with ALS. Further research is warranted to investigate the relationship between non-motor symptoms and disease progression, as well as to develop targeted interventions to improve the quality of life for people with ALS.

## Introduction

Amyotrophic lateral sclerosis (ALS) is a neurodegenerative disease causing progressive weakness of voluntary muscles, with death resulting from neuromuscular respiratory failure, usually 3–5 years after the onset of symptoms (1). There is considerable variation in motor symptoms, which may include walking difficulty, tripping, falling, slurred speech, swallowing problems, struggling with zips or buttons, and muscle twitching (2). There are however other symptoms that patients report, not obviously relatable to motor weakness.

For example, people living with ALS may report pain, autonomic, gastrointestinal, vascular, and psychiatric symptoms (1). These are collectively referred to as non-motor symptoms. Some may however have an origin in motor weakness, making the definition challenging. For clarity, here we define non-motor symptoms of relevance as those with a neurological origin outside the corticobulbar and corticospinal pathways. Such symptoms are important for two reasons. First, they have a significant impact on quality of life, and second, they may have a pre-diagnostic value as endophenotypes, as seen in other neurodegenerative disorders such as Parkinson’s disease.

Because motor symptoms are so prominent in ALS, other symptoms may be under-reported by patients or may be dismissed by physicians. For example, the relationship between frontotemporal impairment or dementia and ALS was unnoticed for many years, until the evidence was made overwhelming by genetic analyses (3–6) even though it was well-recognized in some circles before that (7, 8), and carers would report personality changes to doctors.

A search of studies focused on different aspects of ALS based on publication counts reveals 2210 papers dedicated to the study of non-motor symptoms in ALS, compared with a total of 388,000 publications in ALS on 16/05/2022 (2010–2022, Google scholar). In other words, non-motor symptoms account for only 0.5% of publications, and it is likely that most of these report studies of frontotemporal degeneration and no other non-motor symptoms.

The impact of non-motor symptoms on the daily lives of persons with ALS is comparable to, and in some cases higher, than that of motor symptoms, because of the impact on quality of life (9). We therefore set out to examine the nature and extent of non-motor symptoms in ALS.

## Methods

### Questionnaire development and delivery

To scope the landscape of non-motor symptoms in ALS, we used experience from Parkinson’s disease non-motor symptoms questionnaires, modified after a review of publications on non-motor symptoms in ALS and the anecdotal clinical experience of the authors.

An initial 20-item questionnaire was developed covering the domains of autonomic function, sleep, pain, gastrointestinal disturbance, and emotional lability. The questions are listed in Tables 1–3. Answers were categorized as 0 (never), 1 (sometimes) and 2 (always/nearly always) and the questionnaire was posted through the online system, SurveyMonkey.

**Table 1:** Frequency and association of non-motor symptoms in ALS.

Symptoms	Cases number	Cases percentage	Controls number	Controls percentage	*p*-Value
My skin is dry	324	58.9%	226	41.0%	3.74 × 10^−3^
Things taste different from how they should	149	80.9%	35	19.0%	4.32 × 10^−15^
I can’t help sleeping in the daytime	253	70.8%	104	29.1%	3.22 × 10^−13^
I have lost my appetite	201	81.7%	45	18.2%	1.56 × 10^−21^
I can’t control my feelings when I want	264	71.1%	107	28.8%	2.01 × 10^−15^
I am not happy with my sleep	313	59.7%	211	40.2%	1.60 × 10^−3^
There is a change to my sense of smell	100	80.6%	24	19.3%	2.77 × 10^−10^
I have to get to the toilet quickly when I feel the urge to go	315	73.2%	115	26.7%	4.70 × 10^−23^
I suffer from constipation	279	71.3%	112	28.6%	8.76 × 10^−16^
I sweat a lot	178	66.1%	91	33.8%	7.91 × 10^−6^
I feel nauseous	147	72.0%	57	27.9%	2.00 × 10^−6^
I go to the toilet more often now (urination)	218	68.1%	102	31.8%	2.00 × 10^−6^
I usually suffer from diarrhoea	132	70.5%	55	29.4%	6.90 × 10^−5^
I feel dizzy when I get out of bed	155	70.7%	64	29.2%	8.01 × 10^−6^
I feel dizzy when I turn around	157	70.7%	65	29.2%	8.02 × 10^−6^
I suffer from pain in my neck	297	71.0%	121	28.9%	4.96 × 10^−17^
I suffer from pain in my limbs	322	75.0%	107	24.9%	6.14 × 10^−28^
My limbs are usually cold	360	75.9%	114	24.0%	1.66 × 10^−36^
I feel itchy	260	70.0%	111	29.9%	1.67 × 10^−12^
I do not completely empty my bowels	240	71.8%	94	28.1%	1.60 × 10^−13^

Note: All non-motor symptoms were more frequently seen in ALS than in controls, with some greatly distinguishing the two groups. Percentages show the proportion of cases or controls of the total reporting the symptom.

**Table 2:** Correlation analysis of the responses in people with ALS and controls.

	My skin is dry	Things taste different from how they should	I can’t help sleeping in the daytime	I have lost my appetite	I can’t control my feelings when I want	I am not happy with my sleep	There is a change to my sense of smell	I must get to the toilet quickly when I feel the urge to go	I suffer from constipation	I sweat a lot	I feel nauseous	I go to the toilet more often now (urination)	I usually suffer from diarrhoea	I feel dizzy when I get out of bed	I feel dizzy when I turn around	I suffer from pain in my neck	I suffer from pain in my limbs	My limbs are usually cold	I feel itchy	I do not completely empty my bowels
My skin is dry	1.00	0.20	0.16	0.24	0.12	0.17	0.22	0.16	0.23	0.11	0.26	0.17	0.17	0.17	0.16	0.18	0.19	0.19	0.30	0.26
Things taste different from how they should	0.10	1.00	0.14	0.27	0.18	0.17	0.41	0.15	0.15	0.00	0.24	0.19	0.19	0.20	0.23	0.19	0.15	0.02	0.13	0.25
I can’t help sleeping in the daytime	0.05	0.05	1.00	0.10	0.23	0.29	0.08	0.07	0.09	0.05	0.17	0.15	0.20	0.26	0.23	0.16	0.17	0.09	0.08	0.17
I have lost my appetite	0.09	0.21	0.21	1.00	0.14	0.16	0.28	0.10	0.13	0.07	0.33	0.16	0.07	0.27	0.26	0.17	0.13	0.09	0.17	0.21
I can’t control my feelings when I want	0.00	0.13	0.19	0.24	1.00	0.19	0.19	0.14	0.15	0.06	0.15	0.14	0.00	0.16	0.21	0.12	0.16	0.08	0.10	0.17
I am not happy with my sleep	0.11	0.07	0.16	0.16	0.11	1.00	0.16	0.13	0.10	0.13	0.24	0.14	0.07	0.23	0.21	0.12	0.25	0.13	0.15	0.18
There is change to my sense of smell	0.15	0.51	0.01	0.13	0.13	0.03	1.00	0.12	0.16	0.17	0.25	0.12	0.18	0.22	0.27	0.11	0.16	0.08	0.16	0.19
I must get to the toilet quickly when I feel the urge to go	0.02	0.13	0.21	0.13	0.28	0.17	0.22	1.00	0.16	0.14	0.18	0.49	0.10	0.19	0.15	0.07	0.12	0.13	0.14	0.18
I suffer from constipation	0.13	0.11	0.11	0.19	0.12	0.10	0.03	0.14	1.00	0.16	0.18	0.22	0.00	0.09	0.13	0.13	0.09	0.09	0.14	0.41
I sweat a lot	0.04	0.08	0.18	0.04	0.12	0.13	0.07	0.12	0.01	1.00	0.20	0.10	0.08	0.18	0.12	0.08	0.13	0.09	0.16	0.15
I feel nauseous	0.10	0.18	0.11	0.27	0.15	0.11	0.09	0.05	0.16	0.14	1.00	0.20	0.20	0.36	0.34	0.15	0.13	0.18	0.24	0.29
I go to the toilet more often now (urination)	0.01	0.10	0.24	0.13	0.22	0.13	0.14	0.51	0.10	0.20	0.00	1.00	0.08	0.16	0.14	0.13	0.12	0.09	0.05	0.28
I usually suffer from diarrhoea	0.08	0.14	0.07	0.18	0.10	0.12	0.16	0.18	0.10	0.10	0.27	0.15	1.00	0.19	0.18	0.14	0.14	0.14	0.14	0.16
I feel dizzy when I get out of bed	0.12	0.11	0.29	0.19	0.24	0.12	0.13	0.20	0.08	0.22	0.33	0.18	0.18	1.00	0.65	0.18	0.16	0.13	0.26	0.21
I feel dizzy when I turn around	0.13	0.15	0.21	0.18	0.18	0.06	0.22	0.16	0.06	0.13	0.34	0.12	0.15	0.49	1.00	0.20	0.17	0.11	0.22	0.23
I suffer from pain in my neck	0.17	0.18	0.17	0.22	0.11	0.24	0.23	0.21	0.19	0.12	0.21	0.12	0.15	0.28	0.28	1.00	0.23	0.05	0.13	0.21
I suffer from pain in my limbs	0.04	0.17	0.22	0.13	0.12	0.13	0.21	0.23	0.20	0.25	0.28	0.21	0.24	0.36	0.32	0.33	1.00	0.09	0.19	0.14
My limbs are usually cold	0.13	0.03	0.08	0.21	0.19	0.04	0.10	0.17	0.04	0.16	0.14	0.16	0.16	0.25	0.27	0.21	0.32	1.00	0.14	0.17
I feel itchy	0.19	0.07	0.19	0.16	0.14	0.24	0.10	0.22	0.08	0.17	0.26	0.2	0.22	0.29	0.19	0.21	0.25	0.18	1.00	0.17
I do not completely empty my bowels	0.09	0.14	0.15	0.23	0.20	0.12	0.17	0.31	0.36	0.11	0.20	0.23	0.18	0.23	0.24	0.27	0.28	0.15	0.26	1.00

Note: Shading corresponds to the degree of correlation. Darker colors indicate stronger correlation.

Cases ▪ Controls ▪.

**Table 3:** Multivariate regression analysis of non-motor symptoms in ALS.

Symptoms	Beta	SE	*p*-Value
My skin is dry	−0.135	0.033	4.30 × 10^−5^
Things taste different from how they should	0.117	0.040	3.84 × 10^−3^
I can’t help sleeping in the daytime	0.092	0.032	4.06 × 10^−3^
I have lost my appetite	0.164	0.036	5.00 × 10^−6^
I can’t control my feelings when I want	0.060	0.031	0.00576
I am not happy with my sleep	−0.102	0.032	1.33 × 10^−3^
There is a change to my sense of smell	0.005	0.047	0.908
I have to get to the toilet quickly when I feel the urge to go	0.172	0.035	1.00 × 10^−6^
I suffer from constipation	0.099	0.032	2.36 × 10^−3^
I sweat a lot	−0.009	0.032	0.769
I feel nauseous	−0.020	0.038	0.597
I go to the toilet more often now (urination)	−0.074	0.035	3.22 × 10^−3^
I usually suffer from diarrhoea	−0.017	0.036	0.635
I feel dizzy when I get out of bed	−0.068	0.042	0.107
I feel dizzy when I turn around	−0.053	0.042	0.204
I suffer from pain in my neck	0.084	0.032	8.88 × 10^−3^
I suffer from pain in my limbs	0.172	0.033	3.54 × 10^−7^
My limbs are usually cold	0.270	0.032	3.12 × 10^−16^
I feel itchy	0.071	0.032	0.00283
I do not completely empty my bowels	−0.014	0.035	0.689

Note: ALS was particularly associated with limb coldness, limb pain, dry skin, loss of appetite, and urinary urgency.

A social media platform (Twitter) was used to publicize a link to the questionnaire. To target people with ALS, tweets were made from accounts with high numbers of followers with MND/ALS and with hashtags to increase visibility to those searching for ALS, but so that control responses would be obtained, the invitation explicitly also stated that all were welcome to participate. The questionnaire was made available for a total of 20 weeks from February 2022 to July 2022.

The questionnaire was approved by the Research Ethics Management Application System at King’s College London, reference LRS/DP-20/21-20319.

### Statistical methods

Chi-square statistics were used to compare frequencies of reported symptoms between cases and controls. Correlation analysis was used to identify groups of symptoms.

We built a step-wise multivariable logistic regression model with case-control status as the dependent, and each answer included as a variable. Patients who experienced the symptom regularly or sometimes were compared with those who had never had the symptom.

All analyses were performed in SPSS v21.0 (SPSS Inc, Illinois, USA).

## Results

There were 1018 responses, of which 927 were complete. In the completed questionnaires, there were 506 people with ALS and 421 unaffected individuals declaring themselves as healthy. Because some symptoms were also frequent in controls, the most associated with ALS are not necessarily the most frequent in ALS. Chi-squared analysis showed that loss of appetite, taste, and smell changes were the most frequent symptoms in people with ALS. The most associated symptoms were cold limbs, painful limbs and urinary urgency, but several others were also strongly associated with ALS (Table 1). Diarrhoea, dry skin, nausea, and urinary frequency were the least associated symptoms.

Some symptoms correlated as expected. For example, people with ALS who experienced dizziness while turning also experienced it when they got out of bed. Those with urinary urgency also experienced urinary frequency (Table 2).

Correlation analysis shows both people with ALS and controls exhibit an association between neck and limb pain and also between urinary urgency and urinary frequency, but these relationships are stronger in people with ALS. As might be expected, changes in the senses of taste and smell are also related to one another in both groups. People with ALS who experience dizziness also experience nausea, and controls show the same trend.

Regression analysis showed people with ALS were more likely to report limb coldness, pain, dry skin, loss of appetite, and urinary urgency than controls (Table 3). Additionally, domain-specific logistic regression showed that symptoms in the pain domain were most likely to be reported by people with ALS (Table 4).

**Table 4:** Regression analysis for 5 different domains of non-motor symptoms.

	Beta	SE	*p*-Value
Autonomic domain	0.045	0.011	6.10 × 10^−5^
Sleep domain	−0.032	0.027	0.00243
GI domain	0.067	0.027	0.0128
Pain domain	0.181	0.027	3.72 × 10^−11^
Psychiatric domain	0.173	0.045	1.32 × 10^−4^

## Discussion

We have shown that non-motor symptoms are frequent in people with ALS, with the most commonly reported being urinary urgency, and limb pain with limb coldness, changes to sense of smell and taste, and loss of appetite. Although these are also reported by the control group, all are particularly frequent in ALS, and are important because they are likely to be overlooked and undertreated, yet would be expected to significantly impact quality of life. They may also hold potential as early markers of disease or as biomarkers of prognosis and treatment response (10).

Non-motor symptoms have four broad causes. First, they may derive directly from neuromuscular weakness. For example, sialorrhoea would fall into this category. We have explicitly excluded such symptoms from this analysis because the improvement of the motor state would automatically resolve the symptom. Second, they may derive indirectly from neuromuscular weakness. For example, limb pain from immobility would fall into this category. Third, they may derive from therapies as side effects. Alteration to the sense of taste, for example, might be a consequence of Riluzole use (11). Fourth, and the group we are most interested in, are symptoms derived from a neurological source outside the corticobulbar and corticospinal motor system. Frontotemporal impairment is the most studied (12), but for example, this group would also include any autonomic symptom. For some symptoms, such as constipation or limb coldness, there may be a contribution from two or more sources such as immobility and an autonomic component. Our results do not distinguish between causes of non-motor symptoms and putative non-motor symptoms as part of MND.

Non-motor symptoms are commonly reported (13). More than 80% of our patient population reported having at least one such problem, although the inherent bias in our study design means this is likely to be an over-estimate. Nevertheless, the result is consistent with previous reports (14).

Further research to understand and manage such symptoms is therefore crucial. This observation also suggests that the involvement of neurological structures outside the motor system is the norm for ALS, even disregarding frontotemporal impairment.

Somewhat surprisingly, sleep disturbance and dry skin were the least reported non-motor symptoms in the ALS population surveyed. Indeed, in multivariate analysis, the negative sign for the beta, which indicates a stronger effect for controls than cases, suggests that after taking into account the other non-motor symptoms that might correlate with sleep disturbance and dry skin, they are not major problems for people with ALS.

Correlation analysis shows that non-motor symptoms do tend to cluster. For example, urinary urgency and urinary frequency, as might be expected, tend to correlate. The same for dizziness on turning and dizziness in bed. However, we do see some surprising associations. Dry skin, as expected, correlates with itchiness, but it also correlates with a sensation of incomplete emptying of bowels.

A strength of this study is the large number of people surveyed, which improves confidence in our findings. However, the data source was an online questionnaire, and we had to make the assumption that respondents provided reliable responses, including regarding their diagnosis or health, which we have no way to confirm. Furthermore, we had to keep the questionnaire short because there was no interaction with a researcher who could encourage continued participation, and far more detail is required to understand the full landscape of non-motor symptoms in ALS. In addition, responders were self-selected and therefore represent a biased sample of all potential responders. We have no way of determining the extent of the nature of this bias within the current study design.

In terms of domains, we found that autonomic disturbance, pain, and psychiatric symptoms were most likely to be experienced by people with ALS. The enteric nervous system is currently regarded as a third component of the autonomic nervous system along with sympathetic and parasympathetic systems (15,16). We found all three autonomic components contributed to patient symptoms. Pain is recognized as important in ALS already, despite being understudied, and for example, the MND Association of England, Wales and Northern Ireland has information for patients and healthcare professionals on pain management (17). A recent meta-analysis has shown that pain is frequent, but the detail of its nature is dependent on the questionnaire used for assessment (12). Furthermore, despite its importance to the patient, it is under-reported by patients to health care teams (18). Similarly, a psychiatric component comprising emotional lability and behavioural or cognitive involvement is now accepted as a component of ALS and is well studied.

Despite the growing awareness of non-motor symptoms in ALS, most clinical trials understandably still focus on motor symptoms as the primary outcome. We may be able to better comprehend the pathophysiology of ALS if we consider additional potential underlying mechanisms, thereby improving the quality of life for people with ALS.

## Data Availability

The data that support the findings of this study is available from the corresponding author upon request.
